# Near-Infrared Transcranial Radiation for Major Depressive Disorder: Proof of Concept Study

**DOI:** 10.1155/2015/352979

**Published:** 2015-08-19

**Authors:** Paolo Cassano, Cristina Cusin, David Mischoulon, Michael R. Hamblin, Luis De Taboada, Angela Pisoni, Trina Chang, Albert Yeung, Dawn F. Ionescu, Samuel R. Petrie, Andrew A. Nierenberg, Maurizio Fava, Dan V. Iosifescu

**Affiliations:** ^1^Depression Clinical & Research Program, Massachusetts General Hospital, Boston, MA 02114, USA; ^2^Wellman Center for Photomedicine, Massachusetts General Hospital, Boston, MA 02114, USA; ^3^LiteCure LLC, Newark, DE 19702, USA; ^4^Department of Psychiatry, The Mount Sinai Hospital, New York, NY 10029, USA

## Abstract

Transcranial near-infrared radiation (NIR) is an innovative treatment for major depressive disorder (MDD), but clinical evidence for its efficacy is limited. Our objective was to investigate the tolerability and efficacy of NIR in patients with MDD. We conducted a proof of concept, prospective, double-blind, randomized study of 6 sessions of NIR versus sham treatment for patients with MDD, using a crossover design. Four patients with MDD with mean age 47 ± 14 (SD) years (1 woman and 3 men) were exposed to irradiance of 700 mW/cm^2^ and a fluence of 84 J/cm^2^ for a total NIR energy of 2.40 kJ delivered per session for 6 sessions. Baseline mean HAM-D_17_ scores decreased from 19.8 ± 4.4 (SD) to 13 ± 5.35 (SD) after treatment (*t* = 7.905; *df* = 3; *P* = 0.004). Patients tolerated the treatment well without any serious adverse events. These findings confirm and extend the preliminary data on NIR as a novel intervention for patients with MDD, but further clinical trials are needed to better understand the efficacy of this new treatment. This trial is registered with ClinicalTrials.gov NCT01538199.

## 1. Introduction

*Near-Infrared Radiation: Mechanism of Action*. In experimental and animal models, laser near-infrared radiation (NIR) noninvasively delivers energy to cytochrome c oxidase and by stimulating this key mitochondrial respiratory chain enzyme (COMPLEX IV, electron transfer chain) leads to increased adenosine triphosphate (ATP) production [1–3]. While several NIR wavelengths have been shown to benefit neuronal cell cultures, the most effective ones (830 nm, 670 nm) paralleled the NIR and red action spectra of oxidized cytochrome c oxidase [4]. Data suggest that coherent red light (670 nm diode laser) protects the viability of cell culture after oxidative stress, as indicated by increased mitochondrial membrane potentials [5]. NIR also stimulates neurite outgrowth mediated by nerve growth factor, and this effect could also have positive implications for axonal protection [5]. Neuroprotective effects of incoherent red light, 670 nm light emitting diode (LED) and 630 nm narrow angle LED, have been documented in* in vivo* models of mitochondrial optic neuropathy [6, 7].* In vivo* bioenergetic changes with coherent NIR (810 nm diode laser) were observed at McLean Hospital (Belmont, MA) in beagle dogs, where a shift towards greater bioenergetic efficiency (PCr/*β*-NTP ratio) occurred in the anterior cingulate cortex after transcranial NIR exposure (3 times/week for 2 weeks) (Mintzopoulos et al., unpublished). In animal models of traumatic brain injury (TBI), coherent NIR (810 nm diode laser) appears to be an effective treatment [8–10] and improves neurogenesis [11]. In addition, incoherent NIR exposure (1072 nm LED) has been shown to improve memory performance in middle-aged mice [12].

*Near-Infrared Radiation for Depression and Cognition*. In a double-blind randomized study in healthy volunteers, exposure to coherent NIR (1064 nm laser) significantly improved overall affect, sustained attention, and visual memory [13]. A report of two individual cases and a case series of eleven patients with TBI suggested that exposure to incoherent NIR and red light (870 nm/633 nm LEDs) positively affected sustained attention, memory and executive functions, and self-awareness, self-regulation, sleep, and depressed mood [14, 15]. These benefits in TBI patients were confirmed in a cohort of ten, treated with coherent NIR light (810 nm/980 nm laser) [16]. A preliminary open study in ten depressed subjects has shown that NIR (810 nm LED) may be effective and well tolerated for depression and anxiety despite a history of treatment resistance [17].

Our pilot study aimed at confirming and extending these findings by including a ^31^P-MRS assessment of brain metabolism, as well as safety and efficacy data on repeated administrations of transcranial NIR with a high power laser diode. As an explanatory note to the readers, the authors would like to clarify the circumstances under which our controlled clinical trial (https://www.clinicaltrials.gov/: NCT01538199) only yielded preliminary uncontrolled data on repeated NIR sessions for MDD. The present study was in fact prematurely interrupted by its sponsor, the PhotoThera Inc. PhotoThera had largely invested in three clinical trials (NEST 1, NEST 2, and NEST 3), testing the same NIR device (NeuroThera Laser) used in our study for the treatment of ischemic stroke. NEST 3, the largest of the NEST trials, was stopped due to an interim analysis revealing futility [18]. PhotoThera, financed by venture capitalists, revoked the funding for all its clinical trials, including investigator initiated studies such as ours. PhotoThera closed down in October 2012 and filed for bankruptcy in May 2013.

## 2. Methods

**Figure 1:**
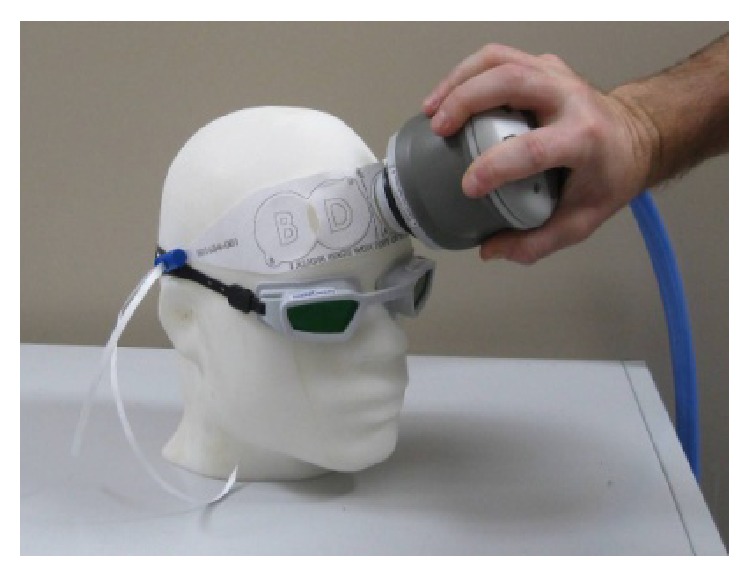
The picture shows the handheld portion of the NeuroThera device, which is pressed against the forehead of the subject. A paper band is used to locate the sites of irradiation across the forehead. Prior to irradiation on each site, the corresponding area of skin is exposed by peeling off the overlying circle of paper from the band.

This single-site study was approved by the Massachusetts General Hospital (MGH) institutional review board (IRB); the Food and Drug Administration (FDA) confirmed that an Investigational Device Exemption (IDE) was not required, as the investigation entailed nonsignificant risks. The Harvard Psychiatry Department (Dupont-Warren Fellowship and Livingston Award), the Brain and Behavior Research Foundation (NARSAD Young Investigator Award), and PhotoThera Inc. sponsored this investigation. Subjects meeting the Diagnostic Statistical Manual-IV (DSM-IV) criteria for MDD, with at least moderate depression (Hamilton depression rating scale, HAM-D_17_ total score between 14 and 24), were included in the study after providing written informed consent. Subjects had failed at most one antidepressant medication and psychotherapy course (stable treatment for at least 6 and 8 weeks, resp.) during the current episode. Active substance use disorders (within prior 6 months), lifetime psychotic episodes, unstable medical illness, and active suicidal or homicidal ideation were exclusionary criteria. Subjects with head-implants, taking light-activated drugs, or having forehead skin conditions (e.g., rash or tattoo) were also excluded. In addition, subjects with implanted metal devices, severe claustrophobia, or weight above 275 lbs were excluded for MRI contraindications. Subjects were randomized to a 7-week double-blind sham-controlled treatment, involving three weeks of either NIR or sham exposures twice a week followed by crossover to three more weeks of the alternate exposure; that is, patients that were exposed to NIR during the first three weeks of the study were exposed to sham, after a washout week, and vice versa. At each treatment session, NIR light (NeuroThera, continuously emitting GaAlAs-laser, manufactured by PhotoThera Inc., with 808 ± 10 nm wavelength (or the same device acting as a sham), with active cooling, total power output 5 W, and verified with a power meter attached to a photodiode detector, Ophir NOVA, prior to the experimental sessions) was administered to the forehead bilaterally at four sites total (2 min per site, 3.3 cm lens piece aperture, 3 cm spot diameter, and 7.1 cm^2^ spot area). We decided to cover the forehead widely (4 sites) to extensively irradiate the prefrontal cortex without overlaps; the data on safety from the NEST 1, NEST 2, and NEST 3 studies on stroke for our same instrument showed that irradiation of up to 20 sites on the scalp was safe (2 min each) [18–21]. Our parameters (including the localization to the forehead) were somewhat consistent with those of Schiffer and colleagues [17], which had demonstrated clinical efficacy of a single session of NIR in depression. NIR was administered with an irradiance of approximately 700 mW/cm^2^ and a fluence of 84 J/cm^2^ (same parameters as in NEST 1, NEST 2, and NEST 3) for a total NIR energy of 2.40 kJ delivered per session with the devices' actively cooled sapphire output lens in direct skin contact. See Table 1 for a summary of the NIR parameters used for this study and comparable studies of transcranial laser therapy (TLT). See Figure 1 for the picture of the handheld portion of the NeuroThera device. No additional information was provided by the manufacturer.

The NeuroThera device was programmed with a list of subjects' treatment codes randomly assigning patients to either NIR or sham exposure for the first three weeks of the crossover study; the device program kept track of the initial assignment and switched the patient's exposure, NIR to sham and vice versa, for the second three weeks of the study treatment. Since the study from Schiffer and colleagues [17] demonstrated that one treatment with NIR was insufficient to determine a durable antidepressant response, we then decided to deliver a course of 6 treatments within 3 weeks. The relatively short timeframe of 3 weeks was decided to allow crossover within an overall 8-week length of trial. The double-blinding was ensured by carrying out identical procedures, identical device interface, including prompts and sounds, and actively cooling the skin surface at the treatment area during both NIR and sham exposures. PhotoThera Inc., the company that manufactured the device, produced data showing that active cooling of the sapphire lenses ensured blinding by preventing skin warming. A specific semistructured scale, the Transcranial Light Therapy Self-Report Questionnaire (T-SR-Q) was completed by participants after the first 6 sessions. The scale explored any discomfort and inconvenience related to the treatment sessions, to detect potential unblinding due to warming effect or to other phenomena. None of the subjects reported skin warming (on the contrary one reported excessive cooling), which validated our blinding. The NIR light is otherwise invisible. All subjects remained on stable antidepressant treatment during the trial. There were no restrictions on class of concomitant antidepressant medications or other concomitant medications, except for photosensitive medications which were exclusionary. Tolerability was assessed with the Systematic Assessment for Treatment Emergent Events (SAFTEE) scale [22]. The primary outcome measures were both change in depressive symptoms (HAM-D_17_) at endpoint and remission from depressive symptoms, defined as HAM-D_17_ ≤ 7. A recent assessment of the interrater reliability among clinicians at our group (MGH Depression Clinical and Research Program) in diagnosing MDD and in measuring the severity of depression has yielded a kappa >0.75. The assessment of interrater reliability was established through live, independent interviews of patients with the use of the HAM-D and SCID-I/P, indicating satisfactory agreement.

We present analysis of four subjects from a sample of eight adults who met study criteria and were initially enrolled in the study. The four subjects excluded from analysis either did not complete the study or, upon review of final study results, failed to meet the study's inclusion/exclusion criteria. One subject dropped out before the first treatment session and two shortly after the first session. These patients could not commit to the full treatment course. One of the patients that dropped out after the first treatment session experienced a headache and left hand paresthesias during the MRS scan, possibly related to preexisting carpal tunnel syndrome. Upon unblinding the device's preprogrammed treatment codes, it was determined that none of the dropouts received active NIR. Data from one more subject were subsequently excluded as her urine repeatedly tested positive for cannabinoids. This latter subject's exclusion was based on her meeting criteria for substance use disorder, which was determined towards the end of her participation. The unblinded treatment codes also showed that all four subjects who completed the 7-week treatment phase and were analyzed received NIR exposures in their first 3 weeks. By means of a paired *t*-test we tested the significance of the change in the mean HAM-D_17_ total score (from baseline) to week 8. Although the primary comparison was with the last assessment (week 8), exploratory analyses were conducted for change from baseline to weeks 4, 5, 6, and 7. A last observation carried forward (LOCF) was also performed to account for one missing value at week 8. We decided not to present the available ^31^P-MRS data since our lack of adequate comparison and our small sample size limited scientific inferences.

## 3. Results

The four completers had a mean age of 47 ± 14 (SD) years (1 woman and 3 men). Their length of depressive episode was at least 18 months. Baseline HAM-D_17_ scores averaged 19.75 ± 4.35 (SD). None of them was taking antidepressant medications at the time of the trial and only one was receiving (long-standing) counseling; however, the counseling was not a proven effective form of psychotherapy for major depressive disorder, such as cognitive behavioral therapy (CBT) or interpersonal therapy (IPT). None of the four subjects had failed to respond to an evidence-based treatment or FDA-approved treatment for depression during the current depressive episode. During the course of the trial, none of the participants started an antidepressant treatment.

**Figure 2:**
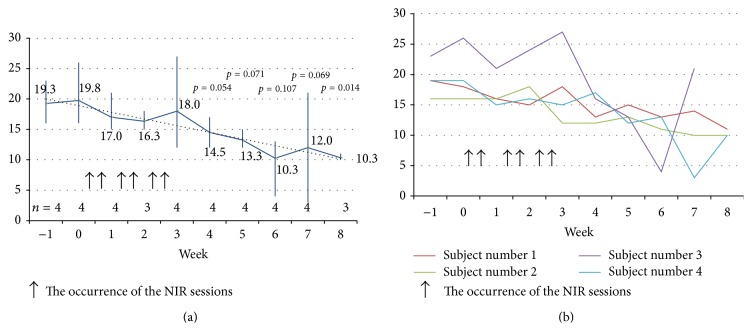
Mean and individual HAM-D_17_ total score ((a) and (b), resp.) for the 4 MDD subjects who sequentially received transcranial NIR and sham. All 4 treatment-completers had received NIR from week 0 to week 3.

Two of the four treatment-completers (50%) achieved remission (albeit not sustained) with HAM-D_17_s of 4 and 3 at weeks 6 and 7, respectively. The mean HAM-D_17_ total score (*n* = 3) decreased from 17.7 ± 1.53 (SD) at baseline to 10.3 ± 0.57 (SD) at week 8 (*t* = 8.315; *df* = 2; *P* = 0.014). On the LOCF analysis, accounting for one missing value at week 8, the mean HAM-D_17_ total score (*n* = 4) decreased from 19.75 ± 4.35 (SD) to 13 ± 5.35 (SD) (*t* = 7.905; *df* = 3; *P* = 0.004).  Figure 2 illustrates the mean HAM-D_17_ total scores over the course of the study for the four subjects. The NIR treatment was well tolerated with no adverse events; only one subject, already diagnosed with irritable bowel syndrome, reported transient, mild diarrhea, which was deemed unrelated to study treatment.

## 4. Discussion

We report findings on the safety and efficacy of multiple NIR sessions in patients with MDD. We observed, in four MDD subjects, that posttreatment depression ratings were significantly reduced from baseline, and the treatment was well tolerated.

Time to remission was 6-7 weeks, consistent with other antidepressant treatments. Since all four subjects who completed the 7-week treatment phase received NIR in their first 3 weeks (Figure 2), a carry-over effect from NIR is likely. In two prior reports, significant clinical and psychological effects were reported as early as week 2, after only a single NIR treatment [13, 17]. A possible explanation for this time to response discrepancy is that NIR in our study was delivered at higher irradiance (700 mW/cm^2^) and fluence (84 J/cm^2^) (Table 1). Alternatively, twice-weekly NIR sessions at four sites for 3 weeks might be suboptimal. A paradoxical dose-response, where higher power and energy densities might be less effective, has been postulated for NIR. Based on animal data, Xuan and colleagues [11] have postulated that an excessive exposure to NIR might be in fact counterproductive. This is somewhat in contrast with other reports in TBI patients, where daily NIR sessions appeared to be beneficial for cognition, although at a much lower NIR fluence (13–20 J/cm^2^) [14, 15]. See Table 1 for the report of eleven cases from Naeser and colleagues [15].

Our 50% treatment remission rate (albeit not sustained) in this small sample size is consistent with the 60% remission rate reported in subjects with resistant MDD treated with NIR by Schiffer and colleagues [17]. Similar to Schiffer and colleagues [17], we used an augmentation design in which NIR was added to stable (at least 6 weeks) treatment. However, because none of our 4 subjects was taking antidepressant medications, we cannot ascribe the high remission rates to synergism. In contrast to Schiffer and colleagues [17], who used incoherent NIR (810 nm), our pilot was conducted with a laser device, coherent NIR. While coherent light (lasers) is likely to achieve deeper penetration into brain tissue [23], we did not observe any apparent advantage by comparing response rates in both studies.

The most important result of this pilot study on repeated NIR treatment is its tolerability and safety in patients with MDD. Our report is the first systematic assessment of tolerability and safety of repeated administration of transcranial NIR with a 5 W laser with high irradiance (700 mW/cm^2^) in depressed subjects. Our finding is consistent with prior case-reports and case series suggesting that multiple transcranial NIR administrations could be safe in TBI subjects [14–16]. We encountered only one potential adverse event characterized by transient and mild diarrhea in a subject with irritable bowel syndrome. This event was attributed to the subject's preexisting medical illness and considered unrelated to the NIR treatment; it resolved within a week and did not require study discontinuation or other interventions. Consistent with our results, several large NIR stroke studies have shown no significant difference in rates of adverse events or serious adverse events between NIR and sham [18, 24].

## 5. Conclusions

Our findings are limited by our small sample size and by a probable carry-over effect. However, they support the hypothesis that TLT could be an effective treatment for MDD. If subsequent studies confirm that a brief course of TLT is effective for treating MDD, this intervention could have advantages over monotherapy with standard antidepressant medications because TLT is noninvasive, has few side effects, and might not require long-term administration. TLT and other treatments targeting brain mitochondria represent an innovative class of therapeutics (compared to standard antidepressant treatment) for patients with MDD.

## Figures and Tables

**Table 1:** Summary of the NIR parameters used for this study (Cassano et al.) and other comparison studies.

	Cassano et al. (this study)	Schiffer et al., 2009 [17]	Barrett and Gonzalez-Lima, 2013 [13]	Naeser et al., 2014 [15]
Study design	Pilot, open	Open	Double-blind randomized NIR versus sham	Open

Subjects enrolled	4 MDD completers	10 resistant MDD	40 psychology undergraduates	11 chronic TBI (8 cases with depression; 5 with at least moderate severity)

Number of treatment sessions	2/week for 3 weeks	1	1	3/week for 6 weeks

Duration of clinical follow-up	8 weeks	4 weeks	2 weeks	14 weeks

Clinical outcome(s)	50% remission of MDD at weeks 6-7 (HAM-D_17_ ≤7)	60% remission of MDD at week 2 (HAM-D-21 <10)	Maintained positive affect at 2 weeks compared to decline of control group (PANAS)	37% response of depression at week 7 (decrease of BDI-II total score ≥50% from baseline)

Device specifications				
Manufacturer/model/type	PhotoThera/NeuroThera/laser diode	Custom device/Marubeni America Corp./LED	Cell Gen Therapeutics/Model CG-5000/laser diode	MedX Health/Model 1100/LED
Wavelength (nm)	808	810	1064	870 and 633
Spot diameter (cm)	3	—	4	5.35
Spot area (cm^2^)	7.1	~1	12.6	22.5

Site exposure parameters				
Time (sec)	120	240	240	585
Irradiance (mW/cm^2^)	700	250	250	22.2
Fluence (J/cm^2^)	84	60	60	13

Treatment exposure parameters				
Number of exposed sites	Four	Two	Two	Eleven
Site locations, transcranial treatments	Bilateral (R and L forehead center at 20 and 40 mm from sagittal line)	Bilateral (R and L forehead at EEG map sites: F3, F4)	Unilateral (R, frontal pole on 4 cm medial and lateral)	Midline and bilateral (forehead (prefrontal) and temporal, parietal, and occipital area)
Total area exposed (“treatment window,” cm^2^)	28.4 (7.1 × 4 sites)	2 (1 × 2 sites)	25.2 (12.6 × 2 sites)	247 (22.5 × 11 sites)
Total energy delivered, per session (kJ)	2.4 (0.6 × 4 sites)	0.12 (0.06 × 2 sites)	1.51 (0.76 × 2 sites)	3.21 (0.29 × 11 sites)
Total energy delivered, per treatment (kJ)	14.4 (total energy per session × 2 sessions/week × 3 weeks)	0.12 (total energy per session × 1 session)	1.51 (total energy per session × 1 session)	57.87 (total energy per session × 3 sessions/week × 6 weeks)
